# Menstrual cycle and hormonal contraceptive phases’ effect on elite rowers’ training, performance and wellness

**DOI:** 10.3389/fphys.2023.1110526

**Published:** 2023-02-17

**Authors:** Juliana Antero, Steven Golovkine, Louis Niffoi, Alice Meignié, Tom Chassard, Quentin Delarochelambert, Martine Duclos, Carole Maitre, Hugo Maciejewski, Allison Diry, Jean-François Toussaint

**Affiliations:** ^1^ Institute for Research in BioMedicine and Epidemiology of Sport, IRMES at INSEP (Institut National du Sport, de l’Expertise et de la Performance), Paris, France; ^2^ Department of Sport Medicine and Functional Exploration, University Hospital CHU G. Montpied, INRAE, UNH, CRNH Auvergne, Clermont Auvergne University, Clermont-Ferrand, France; ^3^ Medical Department at INSEP (Institut National du Sport, de l’Expertise et de la Performance), Paris, France; ^4^ French Rowing Federation, Nogent-sur-Marne, France; ^5^ URP 7329, Université Paris Cité, Paris, France; ^6^ Center for Investigation in Sport Medicine, CIMS Hôtel-Dieu, Assistance Publique—Hopitaux de Paris, Paris, France

**Keywords:** female athletes, menstrual cycle, contraception, hormones and athletes, performance, monitoring, gender

## Abstract

**Objectives:** To investigate the effect of menstrual cycle (MC) and hormonal contraception (HC) phases in elite rowers training, performance and wellness monitoring.

**Methods:** Twelve French elite rowers were follow-up for 4,2 cycles on average in their final preparation for the Olympics and Paralympics Games in Tokyo 2021 through an on-site longitudinal study based on repeated measures. Daily self-reported evaluation using Likert rating scales of wellness (sleep quality, fitness, mood, injuries’ pain), menstrual symptoms and training parameters (perceived exertion and self-assessment of performance) were collected (n = 1,281) in parallel to a coach evaluation of rowers’ performance (n = 136), blinded to theirs MC and HC phases. Salivary samples of estradiol and progesterone were collected in each cycle to help to classify the MC into 6 phases and HC into 2–3 phases depending on the pills’ hormone concentration. A chi-square test normalized by each rower was used to compare the upper quintile scores of each studied variable across phases. A Bayesian ordinal logistic regression was applied to model the rowers’ self-reported performance.

**Results:** Rowers with a natural cycle, n = 6 ( + 1 amenorrhea) evaluate their performance and wellness with significant higher score indices at the middle of their cycle. Top assessments are rarer at the premenstrual and menses phases, when they more frequently experience menstrual symptoms which are negatively correlated with their performance. The HC rowers, n = 5, also better evaluate their performance when taking the pills and more frequently experience menstrual symptoms during the pill withdrawal. The athletes self-reported performance is correlated with their coach’s evaluation.

**Conclusion:** It seems important to integrate MC and HC data in the wellness and training monitoring of female athletes since these parameters vary across hormonal phases affecting training perception of both athlete and coach.

## 1 Introduction

Elite athletes are commonly monitored on a daily basis for training and wellness parameters to follow their readiness and to anticipate training performance and injury risks (Gabbett et al., 2017; Thorpe et al., 2017; Ryan et al., 2020). Standard monitoring systems include daily questionnaires collecting self-reported information on athletes’ fitness, sleep quality, mood, or Rating of Perceived Exertion (RPE) (Haddad et al., 2013) in addition to objective measurements when possible (Anna et al., 2016; Dumortier et al., 2018; Ryan Kempton et al., 2021.

Female sex hormones, notably estrogens and progesterone, fluctuate throughout the natural menstrual cycle (MC) and vary according to the concentration of short-acting hormonal contraception (HC). Hormones may affect multiple parameters on women, ranging from fatigue, sleep disturbance, and mood disorders (excitability, depressive tendency) to altered cardiovascular, muscular and metabolic parameters (Constantini and Gal., 2005; Wang et al., 2020). All these effects may modulate training responses and performance (Oosthuyse et al., 2010). However, there is a lack of studies investigating the effect of the MC phases on the athletes’ monitoring systems. In addition, it is unknown if HC users show differences in these parameters along their hormonal phases (if applicable). It is important to understand if adjustments on monitoring systems, according to elite athletes’ cycle phases would be appropriated, when relying on such parameters to estimate the athletes’ readiness.

Performance in the field context is of great relevance when monitoring elite athletes. Different performance-related parameters are affected during MC, even though the magnitude and the direction of such effects remain unclear (Mcnulty et al., 2020; Meignié et al., 2021; Borja et al., 2022). In addition, most studies have investigated eumenorrheic naturally cycling athletes in a laboratory setting, excluding athletes with irregular cycles or using contraceptive hormones (Meignié et al., 2021), which may not properly document field reality (Martin et al., 2018; Ravi et al., 2021). Also, the performance evaluated in a laboratory setting may not reflect the multifactorial traits of performance and is hardly feasible on a regular basis to monitor elite athletes’ training performance and progression (Meignié et al., 2021). In addition, previous studies showed that MC-related symptoms may be common among elite women athletes (Martin et al., 2018; Findlay et al., 2020), which are often perceived by them as an impairment to their performances (Findlay et al., 2020; Read et al., 2021). However, the effect of MC on daily training performances has not been investigated before. It also remains unclear if there is any correlation between the symptoms felt by the athlete and her training performance on the same day.

Therefore, we aimed to investigate the effect of menstrual cycle and contraceptive hormones phases on training, performance and wellness parameters of elite rowers monitoring using daily self-reported evaluation. We hypothesized that the cycle phases impact the monitored training parameters.

## 2 Methods

### 2.1 Study design

On-field longitudinal study based on repeated measures of an elite rowers’ cohort.

### 2.2 Participants

We asked the French Rowing Federation to select twelve elite female rowers [Elite/International Level according to a previous classification framework (McKay et al., 2021)] to volunteer to this study, mimicking a staff selection inclusion of elite athletes in a monitoring system. The twelve female rowers (ten able-bodied and two paralympic athletes) were informed and volunteered to take part in this study. The athletes were asked to fill in a preliminary questionnaire that collected general information (e.g., age, body mass index, training volume) and their gynecological history (e.g., cycle regularity, contraceptive methods) to identify any contraindications to participate in the study. None presented any gynecological or hormonal condition limiting their participation in the study. Seven athletes had a natural cycle (including two using intrauterine devices) and five used combined contraceptive pills for 21 days (one monophasic, three biphasic and one triphasic) with a 7-day break.

### 2.3 Procedures and monitored parameters

The study started on 14 February 2021, concomitantly with the beginning of the rowers’ competitive season, planned to last three to 6 months. Before the beginning of the follow up, a meeting with the athletes took place to present the protocol. We explained in detail the monitoring they were asked to fill in every morning using a smartphone application created for this study and optimized for minimal burden (Saw and Main., 2015). The questionnaire (see details in the Supplementary Material) included items related to the menstrual cycle, symptoms (Bruinvels et al., 2021) and wellness (sleep duration and quality, fitness, mood, injuries), using Likert rating scales (Haddad et al., 2013; Claudino et al., 2019) ranging from 1 to 10 (where 10 corresponds to top scores). The training sessions were also monitored: the RPE, BORG-CR10 (Borg, 1982; Arney et al., 2019) and Performance (1 = very bad training performance; 10 = excellent training performance). The latter was intended to be a qualitative assessment of the session, dissociated from the RPE (Supplementary Material).

### 2.4 Coaches evaluation of performance

The same Performance evaluation, from 1 to 10, was requested to the three coaches in charge of the 12 athletes enrolled in the study. All three consented to participate in the study. We asked the coaches to evaluate the athlete’s performance after every training session they fully coached, *via* an app exclusively built for the study. Each athlete was evaluated by a single coach. Coaches were blinded to both the athletes cycle phase and the athlete’s answers.

### 2.5 Cycles phase division

**FIGURE 1:**
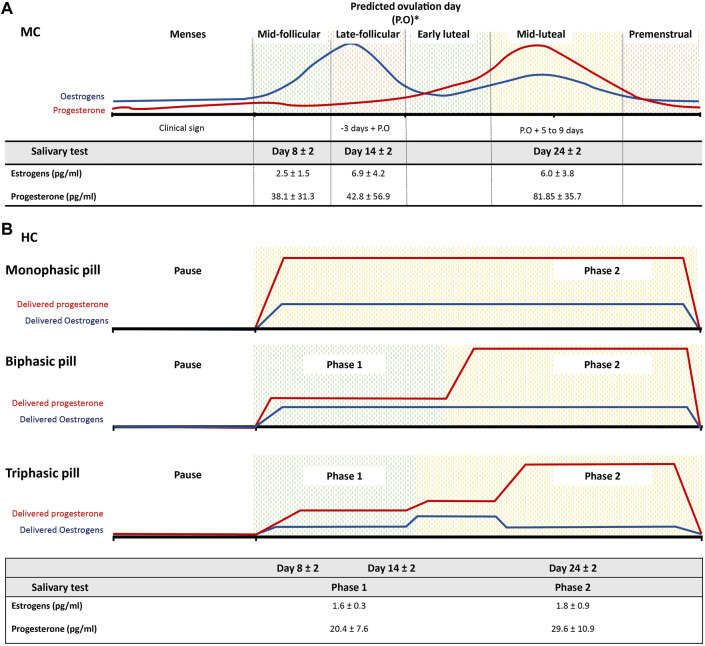
Schematic representation of the phases’ classification among French women elite rowers. **(A)** six phases division for natural menstrual cycle (MC) rowers based on the predicated ovulation day (P.O) and a calendar counting. The table displays the salivary levels (pg/mL) of estrogens and progesterone taken during days 8, 14 and 24 ± 2 days and the corresponding sub-phases following the schematic classification for both MC and **(B)** rowers using hormonal contraception (HC). Two to three phases division according to the type of pills composition for where pause corresponds to the pill withdraw.

The phases of the athletes’ cycles were determined combining the menstrual calendar and the hormones samples. For the naturally MC women, six cycle phases were established based on the predicted day with the highest probability of ovulation (Schaumberg et al., 2017; Soumpasis and Grace., 2020) and a calendar counting as illustrated in Figure 1A.

Salivary samples of estradiol and progesterone were collected at day 8 (mid-follicular), 14 (late follicular) and 24 (mid-luteal) ± 2 days (according to the cycles’ length) of each rowers’ cycle to verify if the hormonal fluctuations were in accordance with our calendar-based phase divisions (Figure 1A). These specific range of days were selected to analyze different hormonal environments as previously suggested (Janse DE Jonge and Thompson., 2019): low sex hormonal levels in the early follicular (in our case we preferred to do after menses, around day 8 since the clinical bleeding allows to precisely identify this cycle phase); low progesterone but high estrogen levels around day 14 and elevated both progesterone and estrogen levels around day 24.

For the HC users, three phases were determined according to their pill composition (Figure 1B). The phase division for both groups is detailed in the Supplementary Material. Salivary samples of estradiol and progesterone were collected as a control of the MC group, that is, at days 8, 14, 24 ± 2 days (counting day 1 as first pill’s pause day) during the pill’s taking for descriptive purpose (Figure 1B).

For a common phase division of the whole group, we used two phases only based on the menstruation status defined by the presence of clinical bleeding.

### 2.6 Data analyses–MC and HC phases

We analyzed the top quintile of each sleep quality, fitness, mood and performance variable normalized by each cycle phase length and by each athlete. Each variable was then categorized in a binary variable, one if the variable of interest on each day is greater than the quantile of order 0.8, that is, the top 20% score, and 0 if it is below. We used a chi-square test of goodness-of-fit with a discrete distribution of parameter 1/k (k: number of phases) to compare the upper quintile distributions of each variable across phases. If the chi-square test was significant at the α = 0.05 threshold, we rejected the hypothesis that the upper quintiles were homogeneously distributed in the phases and analyzed the Pearson residuals to evaluate the phase in which the variable was over- or under-evaluated. If the Pearson residual was greater than $z_{crit}=1.96$ (the quantile of the normal distribution at the threshold 1-α/2), the variable was over-represented in the phase. Conversely, if it was less than $z_{crit}=-1.96\;($ the α/2 quantile), the variable was under-represented in the phase (Haberman., 1973) at the threshold of α = 0.05. The difference at the threshold of α = 0.1 is indicated by a $z_{c}=\pm \;1.64$ .

These analyses were performed according to the use of HC or under a natural MC cycle. Similarly, the frequency of the symptoms was analyzed in these two separated groups and was described as the sum of declared symptoms per phase.

### 2.7 Data analyses–Menses effect in the whole athletes’ group

In order to model the athletes’ self-reported performance and RPE scores in the whole group of rowers, we estimated their probability to note these variables from 1 to 10 according to the menstrual status (yes or no). The estimation of the probabilities was assessed using a Bayesian ordinal logistic regression model BRMS with proportional odds (McElreath, 2020). The model has been fitted with Markov Chain Monte Carlo (MCMC) simulation with 4 chains, 1,000 steps to burn-in and flat priors for the parameters using the “brms” R package (Bürkner, 2017). Convergence of the model has been checked using the Brooks-Gelman-Rubin scale reduction factor (Rhat) with values below 1.1 and by visual inspection of the trace plots of the MCMC chains. The probabilities are reported as the mean of the posterior distribution ± 75% high-density interval.

The coach’s performance evaluation according to the rower’s menstrual status has been analyzed using the same chi-square test method described in the previous section.

A Spearman correlation test was performed to test if the correlation among the studied variables (performance, wellness, sleep duration and bedtime, symptoms, pain intensity and the coach evaluation) were significantly different from zero (α = 0.05).

The baseline characteristics of the studied population were described using the mean (SD), median and proportions. R software was used for all analyses.

### 2.8 Research ethics and data security

Prior to participation, all athletes and coaches were informed about the purpose of the study and the data collection involved in and signed a consent letter. All investigations conformed to the code of ethics of the World Medical Association (Declaration of Helsinki) and were approved by the Institutional Ethics Committee (IRB00012476-2022-03-11-206). Data collection was compliant with the General Data Protection Regulation (2016/679) applied in the European Union and received a certificate of compliancy by the *Comission Nationale Informatique et Libertés* (CNIL -2221532 v0).

## 3 Results

The twelve rowers enrolled in the study were followed for three to 6 months according to their training season (the shortest follow-up ended on May 7th and the longest on July 20th). The longest follow-up corresponds to the five rowers who, during the study, qualified to the Tokyo Summer Olympic or Paralympic Games starting in July or August 2021, respectively.

Athletes were 18–38 years old (22.9 ± 6.2, median = 21.0) and trained six to 7 days a week. During the study, a total of 1,281 daily questionnaires were collected, with an average response rate of 78% (52%–95%). On average, we followed 4.2 full cycles per rower. One athlete with a natural cycle has not declared any menstruation (amenorrhea) during the follow-up and was excluded from further analysis. Therefore, the natural MC group was composed of six rowers and the HC group of five rowers. The coaches evaluated their respective athletes a total of 136 times.

In the MC group, the mean levels of salivary estrogens (pg/mL) were 2.5 ± 1.5 in the mid-follicular, 6.9 ± 4.2 in the late follicular and 6.0 ± 3.8 in the mid-luteal phases. Following the same phases order, the mean levels of salivary progesterone (pg/mL) were 38.1 ± 31.3, 42.8 ± 56.9, 81.85 ± 35.7 (Figure 1A). Different hormonal environments were then observed, with the lower hormonal levels, of both estrogens and progesterone during the mid-follicular phase and the highest hormonal levels found in the mid-luteal phase, while the highest level of estrogens were found in the late follicular phase. In the HC group, the mean levels of salivary estrogens (pg/mL) were 1,6 ± 0.3 in the Phase 1 and 1.8 ± 0.9 in the Phase 2. The levels of salivary progesterone (pg/mL) were, in the same phases order, 20.4 ± 7.6 and 29.6 ± 10.9 respectively (Figure 1B).

### 3.1 Phases effect

**FIGURE 2:**
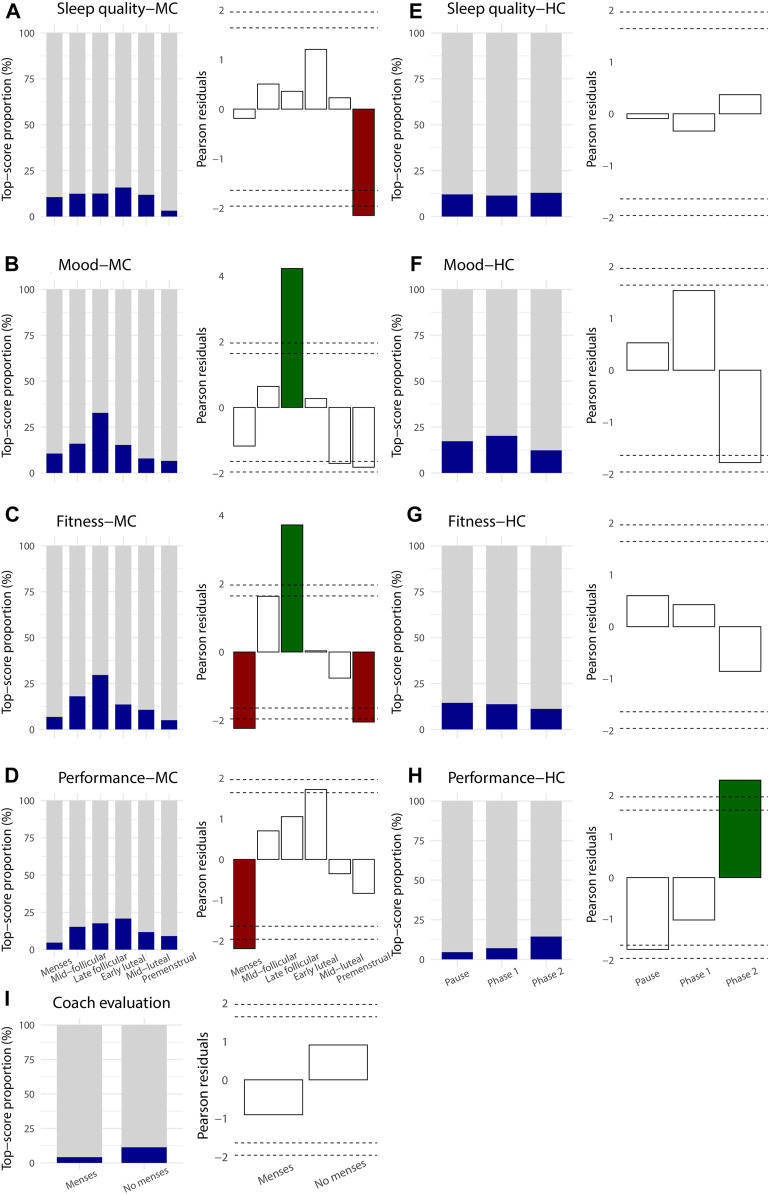
Proportion of the top quintile score of self-evaluation of daily wellness **(A, B, C, D, F, G)** and performance **(D, H)** parameters (dark blue) according to the cycles’ phases in naturally MC rowers (first column **(A–D)**) and hormonal contraception rowers (3^rd^ column **(E–H)**). Pearson residuals of the corresponding top quintile scores in MC (2^nd^ column) and HC rowers (4^th^ column) representing the deviation from the homogeneous distribution of the upper quintiles among the cycles’ phases. Green column: significantly higher proportion of top quintile score ( $z\;>\;z_{crit}$ ); red column: significantly lower proportion ( $z\;<\;z_{crit}$ ). The dotted line at ± 1,96 indicates a Z_c_ at the threshold of α = 0.05 and the dotted line at ± 1.64 indicates a Z_c_ at the threshold of α = 0.1. The I) plot corresponds to the coach’s perception of the athlete’s performance, according to their menstrual status and the corresponding Pearson residuals.

There was a significant phase effect regarding the top quintile individualized self-evaluation related to sleep quality, fitness, mood and performance in the MC group (Figure 2). Among HC users, performance was the only parameter showing significant differences throughout the phases (Figure 2).

In the natural MC group, the proportion of the top quintile self-evaluation of sleep quality was stable along the MC phases, except for the premenstrual phase which showed a significantly lower proportion ( $z\;<\;z_{crit}$ ) (Figure 2A). A top self-reported good mood score was significantly higher during the late follicular phase ( $z\;>\;z_{crit}$ ) and showed a trend of lower score at the end of the cycle ( $z\;<\;z_{c}$ ) (Figure 2B). For the self-reported fitness, the late follicular phase showed the highest proportion of top score ( $z\;>\;z_{crit}$ ) while the premenstrual and menstruation phases showed the lowest ones ( $z\;<\;z_{crit}$ ) (Figure 2C). In the HC group, wellness parameters were stable throughout the phases (Figures 2E–G).

The top quintile of self-reported training performance score in the natural MC group showed a greater but unsignificant proportion in the middle of the cycle (i.e., in the mid follicular, late follicular and early luteal phases ( $z>\;z_{c}$ )) and a significantly lower proportion during the menstruation phase ( $z\;<\;z_{crit}$ ) (Figure 2D). In the HC group, the self-reported performance also showed the lowest proportion of top scores during the pause ( $z\;<\;z_{c}$ ) and the highest ones during phase two ( $z\;>\;z_{crit}$ ) (Figure 2H). The coaches’ perception of athlete performance showed a similar trend with a lower proportion of top scores assessed during the menstrual period (Figure 2I).

**FIGURE 3:**
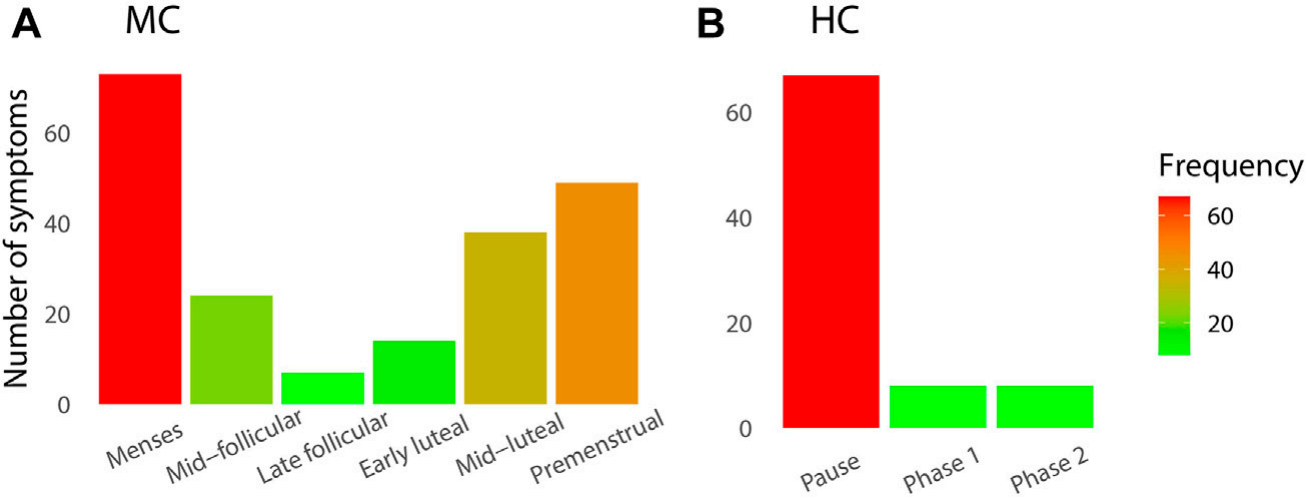
Number of declared symptoms during the longitudinal daily monitoring in elite rowers according to their cycle phases classification for **(A)** natural MC group and **(B)** HC group.

The declared frequency of symptoms was more recurrent during menstruations for both groups (Figure 3). In the natural group (Figure 3A), the end of each cycle (mid-luteal and premenstrual phases) also presented a larger number of symptoms. The late follicular phase showed the least reported symptoms. The most frequent symptoms reported were digestive troubles (15.7%), cramps (11.9%), and headaches (11.2%).

**FIGURE 4:**
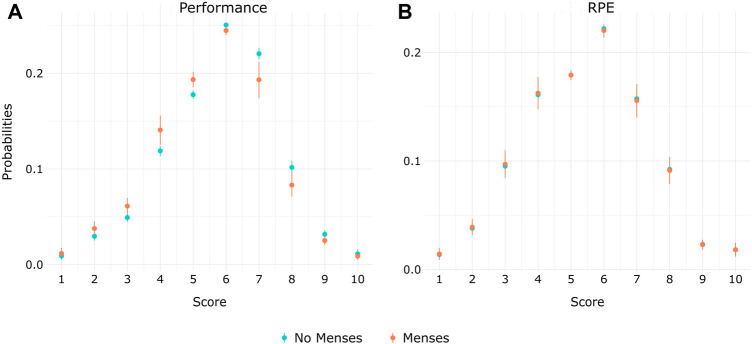
Probabilities among elite rowers of self-evaluating their daily training **(A)** Performance and **(B)** RPE from 1–10 according to the menstrual status (menses/no menses).

When considering the whole group of rowers and the menstruation status, the probability to self-evaluate their training performance with scores below five was higher during the menstruation phase. Conversely, the probability of using scores higher than six was lower during this same phase compared to the rest of their cycle (Figure 4A). No difference of probabilities according to the menstruation status was observed for the RPE scale (Figure 4B).

**FIGURE 5:**
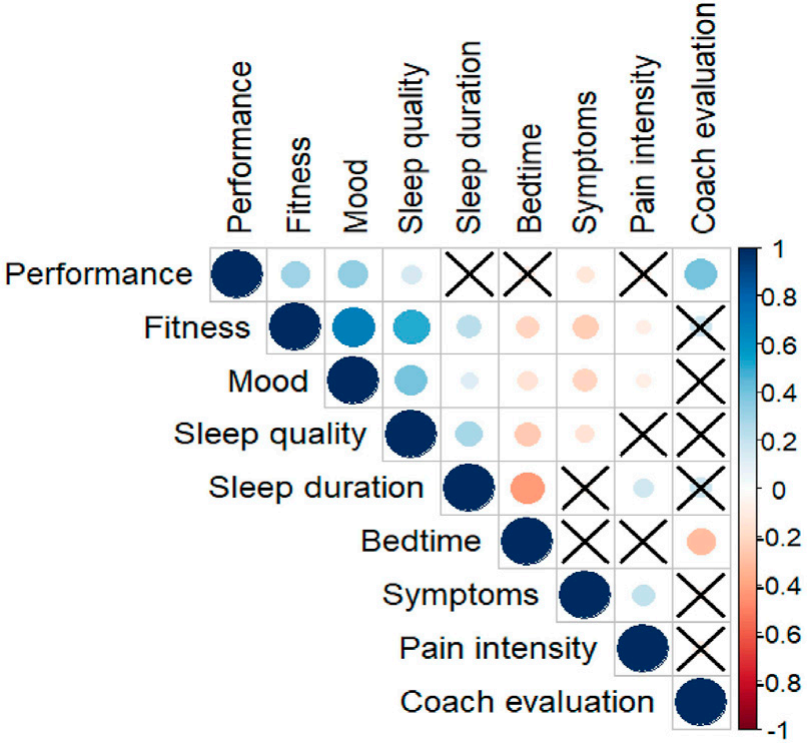
Correlations between the variables monitored during the daily follow-up. A correlation significantly different from zero is indicated by a circle (blue if positive and orange if negative) between a pair of variables. The larger the circle the greater the correlation. A cross indicates a non-significant correlation.

Regarding the significant correlations found among the studied variables, symptoms were negatively correlated with the self-reported training performance (Figure 5). The coaches’ evaluations were correlated with the athletes’ self-reported performances. Wellness parameters-fitness, mood and sleep quality-were also positively correlated with the self-reported performances. Symptoms were negatively correlated with the three wellness parameters and positively correlated with injury pain intensity (Figure 5). Sleep duration and bedtime were not significantly correlated with training performance, but negatively correlated to each other, indicating that when athletes started to sleep late, they did not compensate by waking up later. A late sleep was negatively correlated with the coach’s evaluation. Sleep duration was positively correlated with wellness parameters, while bedtime was negatively correlated to them.

## 4 Discussion

### 4.1 Main findings

Five main findings observed among French elite rowers after three to 6 months of daily on-site monitoring may be highlighted. 1) The self-reported daily performance in training is consistently lower during menstruation among athletes with natural cycle or during pills’ withdrawal among athletes using HC. This self-estimation is correlated with the coaches’ perceptions of performance. 2) During these same phases, menstrual symptoms were most frequent in both MC and HC groups and 3) were negatively correlated with training performance. 4) Sleep quality, fitness and mood fluctuate along the cycle for MC rowers, with a higher proportion of favorable top scores at mid-cycle (around ovulation). 5) Sleep quality, fitness and mood indices are stable throughout HC phases.

Such findings show the importance to assess wellness and training variations across MC or HC phases when monitoring elite female athletes.

### 4.2 Monitoring of subjective variables

In this study, we reproduce what has been used on the field in terms of athletes’ monitoring system (Halson, 2014; Saw and Main., 2015), aiming to evaluate MC and HC phase effects on such monitoring. Up to 91% of high-performance sports employ an athlete monitoring system (Taylor et al., 2012) based on the athlete’s self-reported measurements (Saw and Main., 2015; Thornton et al., 2019). The marked differences between the self-reported performance and RPE in this study model suggest that the athletes precisely distinguished these two scales. The rowers’ training sessions ranges from light aerobic up to maximal effort and is not defined according to their cycle phases (they train together, doing the same program, while their menstrual cycles are not synchronized). Hence, we expected no variations on probabilities of RPEs according to the rowers’ menstrual status, since this measure rather depends on the type of training (Tran et al., 2014) and correlates with objective measures of internal training load (American College of Sports Medicine et al., 2018; Foster et al., 2021). Conversely, the training self-reported performance was intended to be independent of the training program. Our model showed no score difference for the RPE scale according to the menstrual status but revealed differences on the performance score, suggesting a proper use of both scales. In addition, the good correlation between the performance scores provided by the coaches, who are the experts on site, with the athletes’ scores, reinforce the good use of this self-evaluation performance scale.

### 4.3 Self-reported performance and coaches’ expertise

The effect of MC or HC on objective performance measurements remains controversial (Borja et al., 2022). Many reasons have been discussed elsewhere: *i/*the MC effect that accounts for trivial changes only (Mcnulty et al., 2020); *ii/*the limits of testing single points during the cycle rather than the entire cycle *iii/*the limits of relying on menstrual calendar declaration only (Hirschberg, 2022); *iv/*the laboratory testing not reflecting the multiparametric trait of performance (Meignié et al., 2021); *v/*the difficulty to control for potential confounders (e.g., training load, season, recovery, etc.,).

We designed a protocol in order to avoid some of these impediments and focused on daily training performance self-reported assessment. This design provided intra-individual repeated measures (which allows to reduce the impact of potential confounders), taken along the whole cycles (Hirschberg, 2022), using hormone samples to better qualify the phases and avoid misclassification. Yet, the design of this study does not allow any claim of objective and measurable performance changes, only a subjective appreciation from the athletes and their coaches of the work intensity and quality during each training session. However, we believe that the daily performance perceived in the training set is essential as it may impact how the athletes and their coaches anticipate, manage and adapt their training routine. Accordingly, the coaches, despite being blinded to the athletes’ phases, showed a similar trend of a better performance evaluation of their athletes out of their menstruation/pause phase. All these elements contribute to reinforce the finding of a perceived performance change according to the MC or HC phases.

Previous studies investigated the perception of performance during the menses through semi-structured interviews among elite rugby (Findlay et al., 2020) and football players (Read et al., 2021). The majority of the players (ranging from 50 up to 100%) perceived their training and competition performances to be negatively impacted during menses, mostly related to the symptoms perceived. Although such interview method has an important qualitative purpose, it requires a good memory and self-knowledge of one’s cycle relation with training parameters that are multifactorial. In addition, a negative effect is easier to be remembered than a positive one (Baumeister et al., 2001). Overcoming these issues with a daily follow-up, our findings do agree with the results of those studies (although less dichotomic): we showed rarer top-reported performances during menses and more frequent symptoms during this phase, which in turn is negatively correlated with performance. Yet, some top scores were observed during menses. These findings agree with a recent meta-analysis showing a small effect size with a reduced performance during menstruation compared to the other phases (Mcnulty et al., 2020). In addition to what could be called a negative impact during menses, we also showed a positive trend in the middle of the cycle (around ovulation) for the natural MC group and in the phase two of HC users. Especially among natural MC rowers, the observed phase pattern seems to reveal an optimal physical and psychological window occurring around the fertile period. This is important to highlight because when discussing MC with athletes/staff, the discussion is geared towards the potential impairments for female athletes, associated with a negative perception of the menstrual phases (Findlay et al., 2020; Read et al., 2021). We highlighted here a potential cyclic upside in the middle of the MC.

We could also distinguish a cyclicity even among HC. To the best of our knowledge, this is the first study investigating a performance effect (yet subjective) along the use of combined pills.

### 4.4 Wellness parameters

This optimal window in the middle of the cycle (a cycle begins with menses) observed in the natural MC group seems to be the same for fitness and mood parameters, but not for sleep quality.

A study investigating 15 subjective dimensions of mood and energy levels of more than 3 million naturally cycling women found that the menstrual cycle accounted for the greatest magnitude of mood and energy variation compared to daily, weekly and seasonal cycles (Pierson et al., 2021). Similarly to what we observed in the natural MC group of rowers, the lower mood and energy levels evaluation occurs in the beginning and in the end of the cycle, and the highest score in its middle.

We showed a stable evaluation of sleep quality, except in the premenstrual phase in the MC group, which showed rarer top-qualified scores. Such poor sleep quality in the premenstrual phase has been previously documented in ovulatory women in the general population (Driver et al., 2008; Baker et al., 2018). There are also changes in body temperature and effects on subjective and objective measures of sleep, associated with changes in estrogen and progesterone in particular (Moline et al., 2003). Hence, elite athletes seem to be under similar effects of the menstrual cycle of general population women regarding wellness parameters. The effect of MC on these wellness variations may contribute to explain the variations perceived on performance in the natural group.

In contrast, the HC group showed stable wellness parameters across the different phases. This was expected since the hormonal fluctuation, that may affect these parameters, is lower in this group. Here again these data reinforce the suggestion that sexual hormones have similar effects on elite athletes than women in the general population using HC.

### 4.5 Menstrual symptoms

The higher frequency of symptoms during the menses and the pre-menstrual phases in the natural MC group agree with previous studies (Bruinvels et al., 2016). Here, we found comparatively a high frequency of symptoms among the HC users during the pill withdrawal. This could indicate a non-optimal adaptation of the type of pill since HC usually reduces negative menstruation-related symptoms within athletic populations (Martin et al., 2018).

We found a negative correlation between the presence of menstrual symptoms and performance. In addition, we showed that such symptoms are also positively correlated with the intensity of pain perceived when the athletes were injured. The wellness parameters were also found to be diminished in the presence of menstrual symptoms. Hence, the symptoms the athletes perceive could, at least partially, explain the perceived lower performance during menses observed in both groups.

### 4.6 Limits and strengths of this study

In addition to rely on self-reported evaluations, we acknowledge additional limits. First, this study relies on a small sample size refraining an extrapolation of our results to other groups of elite athletes. Secondly, the 78% response rate, while very good for a prospective field study, contains missing data. The athletes reported a simple oversight when asked why they didn’t fulfill the app in the morning and the missing data is rather sparsely distributed along the follow-up, yet, we cannot guarantee that this lack of data is completely random. Also, the coach’s evaluations were scarce because the athletes train very often without the supervision of their main coach. Finally, the phases divisions are approximative and the ovulation day is predicted, not ascertained. Even though we tested the sex hormonal levels in every cycle, this method alone could not detect anovulatory cycles that were probably included in the analysis.

The strengths of this study rely on its design. This was an on-field study with a prospective daily follow-up of a group of high-level athletes in preparation for the Olympic and Paralympic Games 2021. The protocol was intended to adapt to their real training setting and to consider the majority of athletes including those using HC. In addition, we considered the inter-individual differences of each monitored athletes (Burden and Shill., 2021).

### 4.7 Perspective

These findings may impact the athletes monitoring systems common used in elite sports: it shows the importance to integrate menstrual cycle and hormonal contraception in the monitoring of female athletes since they vary across hormonal phases, which affects both the athlete’s and coach’s perception of training performance. The potential cyclic upside in the mid-menstrual cycle and the downside in the premenstrual/menses phases identified may guide sports staff to consider it to anticipate and adapt the training accordingly. The performance variation and the presence of symptoms during the pill withdraw may question the usefulness of such interruption in the elite sport context, for an athlete where a decreased training quality is cyclically identified at individual level during this phase.

This subject should benefit from further investigations on the potential relation between hormones, wellness and training parameters, notably on the response to the training load, that could be tested, preferably through objective measures, on a daily basis.

## 5 Conclusion

French elite rowers with a natural menstrual cycle evaluate their performance and wellness with higher indices at the middle of their cycle. Top assessments are present but much rarer during the premenstrual and menses phases, when they more frequently experience menstrual symptoms. These symptoms are negatively correlated with the perceived performance. The rowers using hormonal contraception also perceive their performance to be better when taking the pills and more frequently experience menstrual symptoms during pill withdrawal. The athletes’ self-reported performance is correlated with their coach’s evaluation.

Our findings suggest the importance to integrate MC and HC phases data in the monitoring systems of female athletes. This might help to better interpret training and wellness parameters, since they vary according to the cycle phases and affect the training perception of both athlete and coach. In addition, the potential cyclic upside in the mid-menstrual cycle and the downside in the premenstrual/menses phases identified may guide sports staff to consider it to anticipate and adapt the training accordingly.

## Data Availability

Data with anonymous and deidentified participant information are available upon reasonable request via protected access to our server.

## References

[B1] Anna E. S. Main L. C. Gastin P. B. (2016). Monitoring the athlete training response: Subjective self-reported measures trump commonly used objective measures: A systematic review. Br. J. Sports Med. 50 (5), 281–291. 10.1136/bjsports-2015-094758 26423706 PMC4789708

[B2] Arney B. E. Glover R. Fusco A. Cortis C. de Koning J. J. van Erp T. (2019). Comparison of RPE (rating of perceived exertion) scales for session RPE. Int. J. Sports Physiology Perform. 14 (7), 994–996. 10.1123/ijspp.2018-0637 30569764

[B3] Baker F. C. Lee K. A. (2018). Menstrual cycle effects on sleep. Sleep. Med. Clin. 13 (3), 283–294. 10.1016/j.jsmc.2018.04.002 30098748

[B4] Baumeister R. Bratslavsky E. Finkenauer C. Vohs K. D. (2001). Bad is stronger than good. Review of General Psychology 5 (4), 323–370. 10.1037/1089-2680.5.4.323

[B5] Borg G. A. (1982). Psychophysical bases of perceived exertion. Med. Sci. sports exerc 14 (5), 377–381. 10.1249/00005768-198205000-00012 7154893

[B6] Borja C. Chang C. J. Watkins R. Senter C. (2022). Optimizing health and athletic performance for women. Curr. Rev. Musculoskelet. Med. 15, 10–20. 10.1007/s12178-021-09735-2 35023069 PMC8804053

[B7] Bruinvels G. Burden R. Brown N. Richards T. Pedlar C. (2016). The prevalence and impact of heavy menstrual bleeding (menorrhagia) in elite and non-elite athletes. PloS One 11 (2), e0149881. 10.1371/journal.pone.0149881 26901873 PMC4763330

[B8] Bruinvels G. Goldsmith E. Blagrove R. Simpkin A. Lewis N. Morton K. (2021). Prevalence and frequency of menstrual cycle symptoms are associated with availability to train and compete: A study of 6812 exercising women recruited using the strava Exercise app. Br. J. Sports Med. 55 (8), 438–443. 10.1136/bjsports-2020-102792 33199360

[B9] Burden R. J. Shill A. L. Bishop N. C. (2021). Elite female athlete research: Stop searching for the ‘magic P. Exp. Physiol. 106 (10), 2029–2030. 10.1113/EP089884 34288150

[B10] Bürkner P-C. (2017). Brms: An R package for bayesian multilevel models using stan. J. Stat. Softw. 80, 1–28. 10.18637/jss.v080.i01

[B11] Claudino J. G. Gabbett T. J. Helton de S. S. Simim M. Fowler P. de Alcantara Borba D. (2019). Which parameters to use for sleep quality monitoring in team sport athletes? A systematic review and meta-analysis. BMJ Open Sport & Exerc. Med. 5 (1), e000475. 10.1136/bmjsem-2018-000475 PMC6340585 30729029

[B12] Constantini N. W. Gal D. Lebrun C. M. (2005). The menstrual cycle and sport performance. Clin. Sports Med. 24 (2), e51–e82. 10.1016/j.csm.2005.01.003 15892917

[B13] Driver H. Werth E. Dijk D. J. Borbely A. A. (2008). The menstrual cycle effects on sleep. Sleep. Med. Clin. 3, 1–11. 10.1016/j.jsmc.2007.10.003

[B14] Dumortier J. Mariman A. Boone J. Delesie L. Tobback E. Vogelaers D. (2018). Sleep, training load and performance in elite female gymnasts. Eur. J. Sport Sci. 18 (2), 151–161. 10.1080/17461391.2017.1389992 29072537

[B15] Findlay R. J. Macrae H. R. E. Whyte Ian Y. Easton C. Forrest née Whyte L. J. (2020). How the menstrual cycle and menstruation affect sporting performance: Experiences and perceptions of elite female rugby players. Br. J. Sports Med. 54, 1108–1113. 10.1136/bjsports-2019-101486 32349965

[B16] Foster C. Boullosa D. McGuigan M. Fusco A. Cortis C. Arney B. E. (2021). 25 Years of session rating of perceived exertion: Historical perspective and development. Int. J. Sports Physiology Perform. 16 (5), 612–621. 10.1123/ijspp.2020-0599 33508782

[B17] Gabbett T. J. Nassis G. P. Oetter E. Pretorius J. Johnston N. Medina D. (2017). The athlete monitoring cycle: A practical guide to interpreting and applying training monitoring data. Br. J. Sports Med. 51, 1451–1452. juin. 10.1136/bjsports-2016-097298 28646100

[B18] Haberman S. J. (1973). The analysis of residuals in cross-classified tables. Biometrics 29, 205–220. 10.2307/2529686

[B19] Haddad M. Chaouachi A. Wong D. P. Castagna C. Hambli M. Olivier H. (2013). Influence of fatigue, stress, muscle soreness and sleep on perceived exertion during submaximal effort. Physiology Behav. 119, 185–189. 10.1016/j.physbeh.2013.06.016 23816982

[B20] Halson S. L. (2014). Sleep in elite athletes and nutritional interventions to enhance sleep. Sports Med. Auckl. N.Z.) 44, S13–S23. 10.1007/s40279-014-0147-0 PMC4008810 24791913

[B21] Hirschberg A. L. (2022). Challenging aspects of research on the influence of the menstrual cycle and oral contraceptives on physical performance. Sports Med. 52, 1453–1456. 10.1007/s40279-021-01616-5 35064914

[B22] Janse De Jonge X. Thompson B. Han A. (2019). Methodological recommendations for menstrual cycle research in sports and Exercise. Med. Sci. Sports Exerc. 51 (12), 2610–2617. 10.1249/MSS.0000000000002073 31246715

[B23] Martin D. Craig S. Cooper S. B. Elliott-Sale K. J. (2018). Period prevalence and perceived side effects of hormonal contraceptive use and the menstrual cycle in elite athletes. Int. J. Sports Physiology Perform. 13 (7), 926–932. 10.1123/ijspp.2017-0330 29283683

[B24] McElreath R. (2020). *Statistical rethinking: A bayesian Course with Examples in R and stan*. New York: Chapman and Hall/CRC.

[B25] McKay A. K. A. Stellingwerff T. Smith E. S. Martin D. T. Mujika I. Goosey-Tolfrey V. L. (2021). Defining training and performance caliber: A participant classification framework. Int. J. Sports Physiology Perform. 17 (2), 317–331. 10.1123/ijspp.2021-0451 34965513

[B26] Mcnulty K. Elliott-Sale K. Dolan E. Paul S. Paul A. Goodall S. (2020). The effects of menstrual cycle phase on Exercise performance in eumenorrheic women: A systematic review and meta-analysis. Sports Med. 50, 1813–1827. 10.1007/s40279-020-01319-3 32661839 PMC7497427

[B27] Meignié A. Duclos M. Carling C. Orhant E. Provost P. Toussaint J-F. (2021). The effects of menstrual cycle phase on elite athlete performance: A critical and systematic review. Front. Physiology 12, 654585. 10.3389/fphys.2021.654585 PMC8170151 34093223

[B28] Moline M. L. Broch L. Zak R. Gross V. (2003). Sleep in women across the life cycle from adulthood through menopause. Sleep. Med. Rev. 7 (2), 155–177. 10.1053/smrv.2001.0228 12628216

[B29] Oosthuyse T. Bosch A. N. (2010). The effect of the menstrual cycle on Exercise metabolism: Implications for Exercise performance in eumenorrhoeic women. Sports Med. Auckl. N.Z.) 40 (3), 207–227. 10.2165/11317090-000000000-00000 20199120

[B30] Pierson E. Althoff T. Thomas D. Hillard P. Leskovec J. (2021). Daily, weekly, seasonal and menstrual cycles in women’s mood, behaviour and vital signs. Nat. Hum. Behav. 5 (6), 716–725. 10.1038/s41562-020-01046-9 33526880

[B31] Ravi S. Waller B. Valtonen M. Villberg J. Vasankari T. Parkkari J (2021). Menstrual dysfunction and body weight dissatisfaction among Finnish young athletes and non-athletes. Scand. J. Med. Sci. Sports 31 (2), 405–417. 10.1111/sms.13838 32979879

[B32] Read P. Mehta R. Craig R. Elena J. Okholm Kryger K. (2021). Elite female football players’ perception of the impact of their menstrual cycle stages on their football performance. A semi-structured interview-based study. Sci. Med. Footb. 0 (0), 616–625. 10.1080/24733938.2021.2020330 36540911

[B33] American College of Sports Medicine Riebe D. Ehrman J. K. Gary L. Magal M. (2018). ACSM’s guidelines for Exercise testing and prescription. Indiana: ACSM. 10.1249/JSR.0b013e31829a68cf 23851406

[B34] Ryan S. Kempton T. Coutts A. J. (2021). Data reduction approaches to athlete monitoring in professional Australian football. Int. J. Sports Physiology Perform. 16 (1), 59–65. 10.1123/ijspp.2020-0083 33152687

[B35] Ryan S. Kempton T. Impellizzeri F. M. Coutts A. J. (2020). Training monitoring in professional Australian football: Theoretical basis and recommendations for coaches and scientists. Sci. Med. Footb. 4 (1), 52–58. 10.1080/24733938.2019.1641212

[B36] Saw A. E. Main L. C. Gastin P. B. (2015). Impact of sport context and support on the use of a self-report measure for athlete monitoring. J. Sports Sci. Med. 14 (4), 732–739. 26664269 PMC4657415

[B37] Schaumberg M. A. Jenkins D. G. Xanne A. K. de Jonge J. Emmerton L. M. Skinner. T. L. (2017). Three-step method for menstrual and oral contraceptive cycle verification. J. Sci. Med. Sport 20 (11), 965–969. 10.1016/j.jsams.2016.08.013 28684053

[B38] Soumpasis I. Grace B. Johnson S. (2020). Real-life insights on menstrual cycles and ovulation using big data. Hum. Reprod. Open 2020 (2), hoaa011. 10.1093/hropen/hoaa011 32328534 PMC7164578

[B39] Taylor K-L. Chapman D. Cronin J. Newton M. Gill N. (2012). Fatigue monitoring in high performance sport: A survey of current trends. J. Aust. Strength Cond. 20, 12–23.

[B40] Thornton H. R. Delaney J. A. Duthie G. M. Dascombe B. J. (2019). Developing athlete monitoring systems in team sports: Data analysis and visualization. Int. J. Sports Physiology Perform. 14 (6), 698–705. 10.1123/ijspp.2018-0169 30676144

[B41] Thorpe R. T. Atkinson G. Drust B. Gregson W. (2017). Monitoring fatigue status in elite team-sport athletes: Implications for practice. Int. J. Sports Physiology Perform. 12, S227–S234. 10.1123/ijspp.2016-0434 28095065

[B42] Tran J. Rice A. J. Main L. C. Gastin P. B. (2014). Development and implementation of a novel measure for quantifying training loads in rowing: The T2minute method. J. Strength Cond. Res. 28 (4), 1172–1180. 10.1519/JSC.0000000000000248 24077376

[B43] Wang Y-X. Arvizu M. Rich-Edwards J. W. Stuart J. J. Manson J. E. Missmer S. A. (2020). Menstrual cycle regularity and length across the reproductive lifespan and risk of premature mortality: Prospective cohort study. BMJ Clin. Res. Ed.) 371, m3464. 10.1136/bmj.m3464 PMC7526082 32998909

