# Supplementary material for: Menstrual cycle and hormonal contraceptive phases’ effect on elite rowers’ training, performance and wellness
Source: Front Physiol. 2023 Feb 17;14:1110526. doi: 10.3389/fphys.2023.1110526 (PMC9981658; doi:10.3389/fphys.2023.1110526)
Supplement: Supplementary file 1 [file DataSheet1.docx]

Supplementary Material

Menstrual Cycle and Hormonal Contraceptive Phases’ Effect on Elite Rowers’ Training, Performance and Wellness

Juliana Antero1, Steven Golovkine1, Louis Niffoi1, Alice Meignié1, Tom Chassard1, Quentin Delarochelambert1, Martine Duclos2, Carole Maitre3, Hugo Maciejewski4, Allison Diry4, Jean-François Toussaint1-6

*** Correspondence:** Corresponding Author: Juliana.antero@insep.fr

# Methods

## Athletes’ daily monitoring questionnaire

In the questionnaire the athletes daily filled in, the beginning and the end of their bleeding period were specified (whether natural or withdrawal bleeding related to the use of HC). They also indicated any possible symptoms, among a list of possibilities (e.g. headache, diarrhea, bloating, menstrual cramps) from previous research [18].

On a scale ranging from 1 to 10 (i.e. Likert scales), athletes answered daily questions regarding their wellness: "How well did you sleep tonight?” ranging from “Very bad sleep quality with insomnia” to “Excellent” [19]; “How do you feel (physically) this morning?” ranging from “Exhausted” to “In top shape” [4] and “Do you have rather positive or negative feelings this morning?” ranging from “Very negative” to “Very positive”. Pairing emotions with their opposites is consistent with previous conceptualizations of emotion [37,43]. The questionnaire also included questions about how much time they slept and the hour they went to bed. When they declared any injury, a pain assessment scale was also required.

Regarding training sessions, athletes were required to evaluate their RPE and Performance. They used a notebook post-session for their coaches and transcript it to the app the next morning. For RPE, we used the RPE scale (BORG-CR10) [20,21]. The training Performance, as we explained to the athletes and coaches prior to the follow-up, is a perception of the quality of the training session and is independent of the effort completed, ranging from 1 (very bad training performance) to 10 (excellent performance at training). Hence, they could have done a light aerobic training but perceived an improvement on their rowing technique for instance; this would refer to a “low RPE” with “high performance”. Likewise, they could have performed a maximal effort and felt they mastered the movement throughout the whole training session; this would be translated as “high RPE” and “high performance” scores. The coach’s evaluation of the athletes’ performance was based on the same Performance Likert scale.

## Natural cycle phases division

To classify the phases in the Natural MC group, a regression model for the follicular phase length was applied, starting on the first day of bleeding up to the predicted day for ovulation [23]. Then this follicular phase was divided into 3 sub-phases [35]: the menses (duration of the bleeding), the mid-follicular, comprised between menses and late follicular and late follicular, which encompasses the estimated ovulation day and the three days before [22,23] (Figure 1a).

The luteal phase length was defined from the end of the late follicular up to the first day of the next bleeding period. We thereafter defined the mid-luteal phase as the 5th to 9th days after the estimated day of ovulation [24]. The luteal phase was then divided into two other sub-phases [35]: early luteal, from the estimated ovulation day up to the beginning of the mid-luteal and the premenstrual phase from the end of the mid-luteal up to the next bleeding day (Figure 1a).

Such 6-phases division was intended to allow us to take in account every training day in a cycle continuous, without classifying in one single phase the potential days were hormonal levels are, at least theoretically, distinct, such is the case of mid-follicular and late follicular, or mid-luteal and early luteal or premenstrual phase. We verified the cycle classification used with the hormone’s level in the mid- follicular, late follicular and mid-luteal phase in order to ensure that hormonal levels differed at least during these keys phases [22]. Not all the 6 phases could be hormonally distinguished nor the ovulation.

## HC phases’ division

For the HC users, three phases were determined according to their composition pill (Figure 1b). The pause phase corresponds to the days without hormonal pills, phase one corresponding to the days with lower hormones concentration (first seven days of the pill pack for biphasic pill and first six for the triphasic pills) and phase two with higher hormone concentration (the last 14 or 15 days).
